# Serological Biomarkers of Intestinal Collagen Turnover Identify Early Response to Infliximab Therapy in Patients With Crohn’s Disease

**DOI:** 10.3389/fmed.2022.933872

**Published:** 2022-07-12

**Authors:** Marta S. Alexdottir, Arno R. Bourgonje, Morten A. Karsdal, Martin Pehrsson, Roberta Loveikyte, Hendrik M. van Dullemen, Marijn C. Visschedijk, Eleonora A. M. Festen, Rinse K. Weersma, Klaas Nico Faber, Gerard Dijkstra, Joachim H. Mortensen

**Affiliations:** ^1^Biomarkers and Research, Nordic Bioscience A/S, Herlev, Denmark; ^2^Department of Biomedical Sciences, Faculty of Health and Medical Sciences, University of Copenhagen, Copenhagen, Denmark; ^3^Department of Gastroenterology and Hepatology, University Medical Center Groningen, University of Groningen, Groningen, Netherlands

**Keywords:** Crohn’s disease, infliximab, TNF-α antagonists, biomarkers, collagen, fibrosis, surgery

## Abstract

**Background:**

Crohn’s disease (CD) is characterized by excessive protease activity and extracellular matrix (ECM) remodeling. To date, 30–50% of patients experience non-response to anti-TNF-α treatment. This study aimed to assess whether serological biomarkers of ECM turnover could monitor or predict response to infliximab (IFX) induction therapy in patients with and without a surgical history.

**Methods:**

Serum biomarkers of type I (C1M), III (C3M), IV (C4M), and VI (C6Ma3) collagen degradation, type III (PRO-C3) and VI (PRO-C6) collagen formation, basement membrane turnover (PRO-C4), and T-cell activity (C4G), were measured at baseline and week 14, in 63 patients with CD undergoing IFX induction therapy. Patients were stratified according to surgical history.

**Results:**

C4M was elevated at baseline in responders with a surgical history (*n* = 10) and associated with response at baseline (*P* < 0.05). Additionally, C6Ma3, PRO-C3, and PRO-C6 were elevated at week 14 in responders compared with non-responders (*n* = 8) and could differentiate between the two groups (*P* < 0.05). Two biomarker ratios (C4M/C4G and PRO-C4/C4G) were elevated at week 14 in non-responders (*n* = 5) without a surgical history compared with responders (*n* = 40) and could differentiate between the response groups (*P* < 0.05).

**Conclusion:**

Baseline levels of a serological biomarker for type IV collagen degradation associated with response to IFX induction therapy, and biomarkers of type III and VI collagen formation may be used to monitor response at the end of induction therapy in patients with a surgical history. Biomarker ratios of type IV collagen turnover demonstrated promising results in monitoring treatment response in patients without a surgical history.

## Introduction

Crohn’s disease (CD) is a type of inflammatory bowel disease (IBD) characterized by prolonged and chronic ulcerative inflammation that can affect any part of the gastrointestinal (GI) tract. Inflammation in CD is believed to be induced by an excessive and inappropriate immune response against the gut microbiota in genetically susceptible individuals (1). Disease pathogenesis is considered to involve a complex interplay of genetic- and environmental factors, gut microbiota, and host immunity (2, 3). Anti-tumor necrosis factor (TNF) agents such as infliximab (IFX) and adalimumab (ADA) have become the mainstay of treatment for patients with moderate-to-severe CD and have proven effective in induction and maintenance of remission (4, 5). However, up to 30% of patients with CD demonstrate non-response or loss of response after induction therapy, leading to longstanding disease activity followed by complications such as fibrotic strictures and penetrating fistulas (6, 7). Although the aim is always to alleviate these disease complications, approximately 70–90% of patients eventually undergo intestinal resection (8). In clinical practice, it remains challenging to predict which patients will respond to anti-TNF induction therapy. Therefore, biomarkers that may aid in predicting response are urgently needed to improve disease management.

The intestinal tissue is rich in extracellular matrix (ECM) proteins such as collagens, which serve an important structural function (9). The intestinal ECM can be divided into two layers: basement membrane (BM) and interstitial matrix (IM). The BM underlies the epithelium and plays an essential role in epithelial barrier integrity, with type IV collagen being the major collagen component – a network-forming collagen that functions as a barrier between tissue compartments. The IM represents the deeper tissue layers and mainly consists of type I and III collagens, both of which are fibrillar collagens that participate in processes such as wound healing, where they act as a scaffold for fibroblast attachment. Type VI collagen can be found in the interface between the BM and IM, separating the two layers. In CD, the ECM remodeling equilibrium is disturbed due to high protease activity, causing injury to the tissue. This results in an influx of inflammatory cells causing BM degradation, which in turn initiates repair processes with increased ECM formation, feeding into the loop of chronic inflammation (10, 11). Proteases involved in ECM remodeling can be expressed by a variety of tissue-resident cells as well as immune cells. For example, granulocytes and macrophages produce matrix metalloproteinases (MMPs) which are known to degrade ECM components, and T-lymphocytes secrete Granzyme-B (GrzB) that enables them to infiltrate through the BM (12, 13).

Fragments derived from intestinal ECM remodeling due to chronic inflammation are released into the circulation and can serve as biomarkers reflecting the pathological disease process (14). These markers could provide personalized treatment and identify changes in the intestinal tissue on a molecular level, adding on another layer of knowledge. Previous studies reported that specific fragments of type III and IV collagen formation and degradation are associated with CD and hold promise as tools for predicting response to anti-TNF therapy (15–17). Additionally, fragments of type I and VI collagen have shown to be elevated in CD patients compared with healthy controls (15, 18). These biomarkers have been validated by various proteomic techniques, as well as in several autoimmune diseases (19–21).

Here we aimed to provide a proof of concept, showing that serological biomarkers of collagen formation and degradation could monitor or predict response to infliximab induction treatment in patients with CD.

## Materials and Methods

### Study Design and Population

This retrospective observational cohort study included patients treated with IFX from February 2011 to December 2018, at the University Medical Center Groningen (UMCG), Netherlands. Diagnosis of CD was based on clinical, endoscopic, and histological criteria. Serum samples were collected on the same day that patients received their first infusion (baseline) and 14 weeks later, which was the time when the response to therapy was evaluated. At the time of sampling, detailed phenotypic data were collected for all patients: Montreal disease classification, medication use, history of bowel surgery, disease activity, and standard laboratory parameters. The inclusion criteria for this study consisted of: an established CD diagnosis existing for at least one year, age ≥18 years, and having an active disease based on a combination of clinical scores and biochemical measures (e.g., CRP, fecal calprotectin, or both) as an indication for starting IFX therapy. Exclusion criteria were as follows: patients undergoing any major surgery or endoscopic balloon dilatation <6 months of sampling, patients with concurrent malignancies (except for skin cancer and hematological malignancies), other fibrotic diseases (e.g., liver fibrosis/cirrhosis, and lung fibrosis), concurrent infections, or perianal disease as the only indication for starting induction therapy with IFX. Clinical disease activity was assessed using the Harvey-Bradshaw Index (HBI) (where active disease was defined as an HBI ≥5) and Physician’s Global Assessment (PGA). Clinical response to treatment was defined as a composite of clinical response (based on HBI and PGA) and steroid-free remission at week 14. This study was approved by the Institutional Review Board (IRB) of the UMCG (IRB no. 08/338). All patients provided written informed consent for the use of patient data and serum. The study was conducted according to the principles of the Declaration of Helsinki (2013).

### Biomarker Assays

Biomarkers for neo-epitope fragments of ECM formation and degradation were measured using solid-phase competitive enzyme-linked immunosorbent assays (ELISAs). The assays were either colorimetric or chemiluminescent. The biomarkers included in this study and references to their technical papers, where information on validation parameters and detailed assay conditions can be found, are listed in Table 1. In addition, three biomarker ratios were computed and included in the study: C3M/PRO-C3 (reflecting type III collagen turnover), PRO-C4/C4G (reflecting the balance between BM turnover/T-cell activity), and C4M/C4G (reflecting the balance between type IV collagen myeloid-/lymphoid cell-mediated degradation). A total of 100 μl of biotinylated antigen in assay buffer was added to 96-well streptavidin-coated plates (Roche Diagnostic cat. no. 11940279, Hvidovre, Denmark) and incubated for 30 min at 20°C while shaking at 300 rpm. After each incubation step, plates were washed with washing buffer (25 mM TRIZMA, 50 mM NaCl, 0.036% Bronidoz L4, 0.1% Tween 20) using a standardized ELISA plate washing machine (BioTek Instruments, Microplate washer, Elx405 Select CW). Standards, controls, and serum samples (20 μl/well) were diluted appropriately and added to the plate, followed by the addition of a horse-radish peroxidase-conjugated monoclonal antibody (100 μl/well) specific for each neo-epitope fragment being measured. The plates were then incubated at either 20°C for 1 h or 4°C overnight, depending on the biomarker. For the chemiluminescence assays (C4G, C6Ma3), 100 μl of BM Chemiluminescence ELISA Substrate (Merck, CAT no. 11582950001) was added to each well and incubated for 3 min, with 1 min of shaking at 300 rpm. Subsequently, the plates were read using a fluorescent plate reader (Fluroskan FL, Thermo Fisher), with a read light emission at 1,000 ms and no filter. For colorimetric assays (C1M, C3M, PRO-C3, C4M, PRO-C4, PRO-C6), 100 μl of tetramethylbenzidine (TMB) (Kem-En-Tec, CAT no. 438OH, Taastrup, Denmark) was added per well and incubated for 15 min at room temperature while shaking at 300 rpm. The reaction was stopped by adding 100 μl of stop solution (0.1 M H_2_SO_4_) per well. The optical density (OD) was measured at 450 nm with 650 nm as a reference, using a VersaMax ELISA microplate reader (Molecular Devices LLC). Finally, standard curves were plotted using 4-parameter logistic models.

**TABLE 1:** Serum biomarkers of extracellular matrix formation/degradation, intestinal inflammation, and immune cell activity.

Protein	Biomarker of degradation	Biomarker of formation	Reflective of	References
Type I collagen	**C1M**: neo-epitope of MMP-2, -9, -13 mediated degradation of type I collagen	–	IM degradation	(34)
Type III collagen	**C3M**: neo-epitope of MMP-9 mediated degradation of type III collagen	**PRO-C3**: released N-terminal pro-peptide of type III collagen	IM turnover	(19, 35)
Type IV collagen	**C4M**: neo-epitope of MMP-2, -9, -12 mediated degradation of type IV collagen alpha 1 chain **C4G**: neo-epitope generated by T-cell granzyme-B-mediated degradation of type IV collagen	**PRO-C4**: internal epitope in 7s domain of type IV collagen	BM turnover	(36–38)
Type VI collagen	**C6Ma3**: MMP-2 and -9 degraded type VI collagen	**PRO-C6**: C-terminal released C5 domain of type VI collagen α3 chain	BM/IM degradation	(39, 40)

*BM, basement membrane; IM, interstitial matrix; MMP, matrix metalloproteinase.*

### Statistical Analysis

All data were considered non-parametric after visual assessment of normality using density plots. Baseline characteristics of the study population are presented as medians with interquartile ranges (IQR) or as proportions *n* with corresponding percentages (%). Differences in demographic and clinical data were compared using Mann–Whitney *U*-tests for continuous variables, and Fisher’s exact tests for factor variables. Univariable logistic regression analysis (method: enter) was applied to identify variables significantly associated with response to treatment. A logistic regression model with interaction terms was subsequently used to evaluate whether the relation between serum biomarker levels (as explanatory variables) and response to treatment (as outcome variable) was dependent on a history of prior surgery (interaction variable), which was identified as a relevant confounding factor. To test the significance of the interaction, likelihood-ratio tests were performed to compare the interaction model to an additive logistic regression model in which history of prior surgery was included as an explanatory variable. Serum biomarker levels were presented as medians with IQR, and differences between groups were tested non-parametrically using Mann–Whitney *U*-tests. Longitudinal differences between biomarker levels from baseline to week 14 were presented as median (IQR). Receiver operating characteristics (ROC) statistics with the area under the curve (AUC) as an overall measure of fit were used to assess the discriminative capacity of the biomarkers. Sensitivity and specificity metrics were obtained by determining the optimal cut-off using Youden’s *J* statistic. AUC values were presented with their corresponding 95% confidence intervals (CI). Statistical analyses were performed using R version 4.1.2. (Rstudio, Boston, MA, United States). Data visualization was performed using Rstudio (version 4.1.2; Rstudio, Boston, MA, United States) and GraphPad Prism 9.2.0 (GraphPad software, San Diego, CA, United States). *P*-values < 0.05 were considered statistically significant.

## Results

### Cohort Characteristics

The baseline demographic and clinical characteristics of the study cohort are shown in Table 2. The median patient age was 35 years [IQR: 26–45] and the majority (57%) were male. A significant difference was noted in disease activity based on the HBI score, where 74% of responders were in clinical remission (HBI <5) at baseline compared with only 27% of non-responders (*P* = 0.019). Most of the patients had either ileal (38%, Montreal L1) or ileocolonic (43%, Montreal L3) disease. Regarding disease behavior, 48% of patients had non-stricturing/non-penetrating disease, while 24% had stricturing disease, and 28% had penetrating disease. In total, 18 patients (29%) had a history of either ileocecal resection, colectomy, or both (Table 2). Surgical history was identified as a relevant confounding factor in logistic regression analyses, showing a considerable impact on biomarker levels with significantly elevated levels of type I, III, IV, and VI collagen degradation in patients without surgical history (Supplementary Figure 1 and Supplementary Table 1). Patients were stratified according to the history of prior surgery in order to prevent distortion of the association between biomarker levels and response to treatment. Baseline demographic and clinical characteristics of the study population separated by surgical history are shown in Supplementary Table 2.

**TABLE 2:** Baseline demographics and clinical characteristics of the study cohort.

	Total cohort *n* = 63	Non-responders *n* = 13	Responders *n* = 50	*P* (non-responders vs. responders)
Age (years)	35 [26, 45]	47 [42, 52]	29 [25, 42]	<0.001
Gender				0.719
Female	27 (43%)	5 (38%)	22 (44%)	
Male	36 (57%)	8 (62%)	28 (56%)	
BMI (kg/m^2^)	24.6 [22.1, 29.1]	28.3 [21.6, 29.9]	24.3 [22.2, 28.8]	0.425
Smoking				0.202
No	20 (32%)	3 (23%)	17 (34%)	
Previous	24 (38%)	8 (62%)	16 (32%)	
Current	19 (30%)	2 (15%)	17 (34%)	
**Montreal classification**				
Montreal age (A)				0.006
A1 (≤16 years)	8 (13%)	1 (7.7%)	7 (14%)	
A2 (17–40 years)	45 (71%)	6 (46%)	39 (78%)	
A3 (>40 years)	10 (16%)	6 (46%)	4 (8.0%)	
Montreal location (L)				0.650
L1 (ileal disease)	24 (38%)	7 (54%)	17 (34%)	
L2 (colonic disease)	5 (7.9%)	1 (7.7%)	4 (8.0%)	
L3 (ileocolonic disease)	27 (43%)	4 (31%)	23 (46%)	
L4 (upper GI disease)	7 (11.1%)	1 (7.7%)	6 (12%)	
Montreal Behavior (B)				>0.999
B1 (non-stricturing, non-penetrating)	30 (48%)	6 (46%)	24 (48%)	
B2 (stricturing)	15 (24%)	3 (23%)	12 (24%)	
B3 (penetrating)	18 (28%)	4 (31%)	14 (28%)	
Montreal perianal disease (P)	17 (27%)	2 (15%)	15 (30%)	0.485
**Medication use, *n* (%)**				
Aminosalicylates	4 (6.3%)	2 (15%)	2 (4.0%)	0.186
Steroids	18 (29%)	7 (54%)	11 (22%)	0.038
Immunosuppressives	49 (78%)	9 (69%)	40 (80%)	0.461
Prior anti-TNF-α	15 (24%)	4 (31%)	11 (22%)	0.146
Prior vedolizumab	1 (1.6%)	1 (7.7%)	0 (0%)	0.206
**Surgical history**				
Colectomy	3 (4.8%)	2 (15%)	2 (4.0%)	0.186
Ileocecal resection	15 (24%)	7 (54%)	8 (16%)	**0.009**
Both	1 (1.6%)	1 (7.7%)	0 (0%)	N/A
**Clinical disease activity score**				
HBI^†^				**0.019**
Remission (<5)	26 (62%)	3 (27%)	23 (74%)	
Mild disease (5–7)	6 (14%)	3 (27%)	3 (9.7%)	
Moderate disease (8–16)	10 (24%)	4 (36%)	4 (13%)	
Severe disease (>16)	2 (4.8%)	1 (9.1%)	1 (3.2%)	
**Laboratory parameters**				
Hemoglobin (nmol/L)	8.05 [7.40, 8.40]	8.20 [7.40, 8.50]	7.90 [7.40, 8.30]	0.808
WBC (× 10>^9^/L)	7.60 [6.00, 9.55]	9.30 [6.90, 10.30]	7.15 [5.85, 8.95]	0.218
Neutrophil count (× 10^9^/L)	5.51 [3.97, 6.81]	6.42 [4.56, 7.52]	5.30 [3.91, 6.56]	0.364
Eosinophil count (× 10^9^/L)	0.12 [0.08, 0.17]	0.14 [0.07, 0.25]	0.11 [0.08, 0.16]	0.574
CRP (mg/L)	5 [2, 12]	3 [1, 9]	6 [3, 12]	0.213
Creatinine (μmol/L)	64 [58, 80]	66 [58, 69]	64 [59, 80]	0.791
eGFR (mL/min/1.73 m^2^)	108 [92, 122]	105 [88, 107]	111 [94, 125]	0.067
Fecal calprotectin (μg/g)^∧^	1.110 [348, 2.415]	1.170 [740, 2.980]	958 [321, 2.195]	0.551

*Data are presented as proportions n with corresponding percentages (%) or medians with IQR in the case of continuous variables. ^†^Clinical disease activity scores (HBI) were available for n = 44 (70%) patients. ^∧^Fecal calprotectin levels were available for n = 23 (37%) patients. The bold values are meant to highlight the significant difference between patients both in clinical parameters and biomarker levels.*

### Baseline Serum Levels of Type IV Collagen Degradation Are Associated With Infliximab Treatment Response in Patients With a Surgical History

**FIGURE 1:**
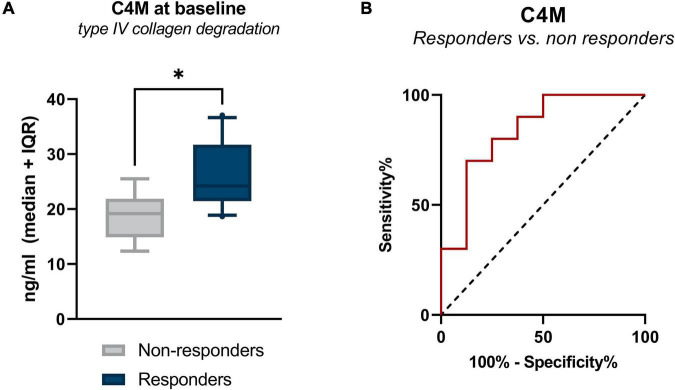
**(A)** Serum levels of C4M in CD patients with surgical history, stratified by response to IFX treatment. Baseline levels of type IV collagen degradation (C4M) were elevated in responders compared with non-responders (*P* < 0.05). **(B)** Receiver operating characteristic curve showing the discriminative power of C4M at baseline to differentiate between responders and non-responders at week 14 (*P* = 0.016). *P < 0.05.

Serum concentrations of all biomarkers measured in patients with CD with surgical history, stratified by response to IFX are presented in Table 3. Baseline levels of MMP-mediated collagen type IV degradation (C4M) were elevated in patients with a surgical history who responded to treatment compared with those who did not respond (C4M: 24.23 ng/ml [22.13, 29.78] vs. 19.20 ng/ml [16.53, 21.68], *P* < 0.05) (Figure 1A and Table 3). ROC statistics revealed that C4M was able to accurately discriminate response from non-response to IFX treatment at baseline in patients with surgical history (AUC with 95% CI: C4M 0.84 [0.64–1.0], *P* = 0.016) (Figure 1B and Supplementary Table 3).

**TABLE 3:** Serum biomarker levels of the cohort stratified by history of surgery and further by response to treatment, both at baseline and week 14.

	History of prior surgery (yes-surgery)			No history of prior surgery (no-surgery)		
	Non-responders (*n* = 8)	Responders (*n* = 10)	*P*-value	Non-responders (*n* = 5)	Responders (*n* = 40)	*P*-value
**Baseline**						
*C1M*	36.49 [28.25, 41.18]	41.13 [35.36, 58.32]	0.286	105.94 [68.52, 126.94]	56.16 [33.36, 101.09]	0.139
*C3M*	9.63 [7.77, 10.58]	10.54 [9.23, 12.54]	0.248	12.58 [11.58, 12.71]	11.71 [9.86, 14.12]	0.448
*C3M/PRO-C3*	1.20 [0.99, 1.47]	1.32 [0.91, 1.36]	0.929	1.49 [1.25, 2.14]	1.36 [1.14, 1.88]	0.348
*C4G*	18.11 [13.48, 24.22]	19.01 [14.25, 28.97]	0.477	16.51 [16.12, 16.71]	17.02 [13.85, 24.08]	0.613
***C4M***	**19.20 [16.53, 21.68]**	**24.23 [22.13, 29.78]**	**0.016**	25.29 [23.07, 32.81]	27.79 [23.61, 33.05]	1.000
*C4M/C4G*	1.11 [0.92, 1.23]	1.38 [0.95, 1.70]	0.477	1.53 [1.29, 1.96]	1.49 [1.15, 1.98]	0.588
*C6Ma3*	0.48 [0.44, 0.50]	0.51 [0.49, 0.84]	0.110	0.66 [0.59, 0.70]	0.60 [0.46, 0.84]	0.759
*PRO-C3*	7.85 [6.01, 9.53]	9.22 [7.67, 10.36]	0.374	8.52 [5.88, 8.84]	8.34 [7.02, 10.60]	0.348
*PRO-C4*	143.96 [121.93, 168.88]	165.00 [147.41, 198.97]	0.131	169.04 [163.80, 191.24]	178.82 [117.99, 234.12]	0.639
*PRO-C4/C4G*	8.04 [6.32, 8.82]	10.39 [5.78, 11.36]	0.534	9.92 [9.68, 10.12]	9.18 [6.75, 13.24]	0.367
*PRO-C4/C4M*	7.45 [7.06, 7.79]	6.76 [6.27, 7.73]	0.477	6.61 [6.48, 7.52]	6.63 [5.71, 6.92]	0.588
*PRO-C6*	6.93 [5.96, 7.63]	9.44 [7.63, 14.13]	0.051	7.78 [6.31, 8.04]	7.41 [6.15, 8.99]	0.718

	**Non-responders (*n* = 8)**	**Responders (*n* = 7)**	***P*-value**	**Non-responders (*n* = 5)**	**Responders (*n* = 40)**	***P*-value**

**Week 14**						
*C1M*	27.83 [23.97, 28.46]	34.56 [23.11, 40.11]	0.271	55.41 [47.90, 62.92]	45.97 [25.33, 70.28]	0.647
*C3M*	8.90 [8.35, 10.97]	9.93 [7.70, 10.62]	0.908	12.97 [12.44, 16.20]	11.83 [10.51, 14.58]	0.225
*C3M/PRO-C3*	1.17 [1.02, 1.81]	0.77 [0.69, 0.98]	0.064	1.4 4[1.14, 2.17]	1.28 [0.97, 1.63]	0.598
*C4G*	18.02 [13.26, 22.31]	16.36 [12.45, 23.98]	0.817	13.58 [12.72, 14.87]	18.02 [14.21, 21.64]	0.114
*C4M*	18.66 [17.81, 20.42]	21.47 [17.97, 26.30]	0.355	26.87 [26.74, 41.37]	26.86 [23.50, 31.98]	0.343
***C4M/C4G***	1.05 [0.80, 1.44]	1.75 [0.86, 2.21]	0.418	**2.25 [2.11, 2.85]**	**1.52 [1.09, 1.90]**	**0.035**
***C6Ma3***	**0.49 [0.46, 0.54]**	**0.62 [0.54, 0.80]**	**0.032**	0.76 [0.63, 0.93]	0.65 [0.53, 0.74]	0.399
***PRO-C3***	**7.59 [6.38, 8.48]**	**10.99 [10.79, 13.39]**	**0.004**	9.02 [7.86, 11.59]	9.50 [8.19, 11.50]	0.752
*PRO-C4*	142.34 [125.24, 158.87]	153.76 [120.06, 178.10]	0.643	272.04 [218.82, 358.82]	185.56 [140.66, 222.74]	0.188
***PRO-C4/C4G***	6.72 [6.10, 10.02]	12.02 [4.98, 14.05]	0.728	**20.04 [17.00, 23.80]**	**10.18 [5.77, 13.01]**	**0.020**
*PRO-C4/C4M*	7.34 [6.47, 7.94]	6.32 [6.12, 7.98]	0.817	7.97 [7.10, 9.05]	6.80 [5.88, 7.31]	0.246
***PRO-C6***	**6.97 [6.49, 7.39]**	**11.04 [10.08, 16.52]**	**0.037**	7.32 [6.47, 7.42]	7.69 [6.81, 9.32]	0.292

*Data are presented as medians with inter quartile ranges (IQR). The bold values are meant to highlight the significant difference between patients both in clinical parameters and biomarker levels.*

### Serum Levels of Type III and VI Collagen Formation and Type VI Collagen Degradation Can Monitor Infliximab Treatment Response in Patients With a Surgical History

**FIGURE 2:**
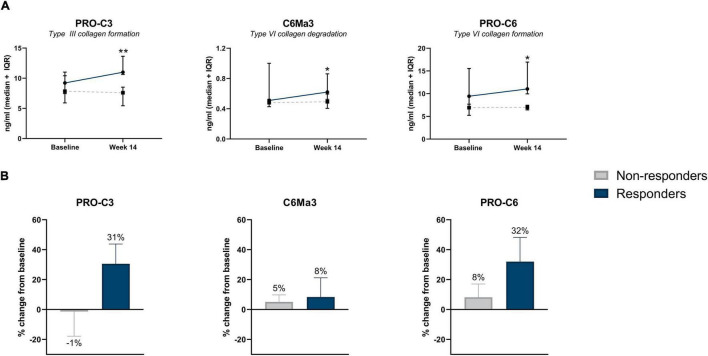
**(A)** Serum biomarker levels in CD patients with surgical history, stratified by response to IFX treatment. Serum levels of collagen type III formation (PRO-C3), type VI degradation (C6Ma3), and type VI formation (PRO-C6) were elevated at week 14 in patients responding to treatment compared with non-responders (*P* = 0.004, *P* = 0.032, *P* = 0. 037). Data are shown as median + IQR. **(B)** Percent change from baseline of type III collagen formation, type VI collagen degradation and formation between responders and non-responders within CD patients with history of prior surgery. *P < 0.05; **P < 0.01.

Increased concentrations of type III collagen formation (PRO-C3), type VI collagen degradation (C6Ma3), and type VI collagen formation (PRO-C6) were observed at week 14, with levels being higher in patients responding to the treatment compared with patients who did not respond (PRO-C3: 10.99 ng/ml [10.79, 13.39] vs. 7.59 ng/ml [6.38, 8.48], *P* < 0.01; C6Ma3: 0.62 ng/ml [0.54, 0.80] vs. 0.49 ng/ml [0.46, 0.54], *P* < 0.05; PRO-C6: 11.04 ng/ml [10.08, 16.52] vs. 6.97 ng/ml [6.49, 7.39], *P* = 0.037) (Table 3 and Figure 2A). ROC analysis showed that these biomarkers could accurately differentiate between responders and non-responders at week 14 (AUC with 95% CI: PRO-C3 0.95 [0.83–1.00] *P* = 0.004; C6Ma3 0.83 [0.62–1.00], *P* = 0.032; PRO-C6 0.82 [0.58–1.00], *P* = 0.037) (Supplementary Table 3). The relative (%) change in PRO-C3 and PRO-C6 levels compared to baseline was +31 and +32% for responders compared to −1 and +8% for non-responders. Little to no difference was observed in change from baseline of C6Ma3 between responders and non-responders (Figure 2B). Although not significant, a trend was seen in type I, III, and IV collagen degradation markers where a clear separation was seen in biomarker levels between non-responders and responders, with responders having higher levels both at baseline and week 14 (Table 3).

### Biomarkers of Type IV Collagen Remodeling Can Monitor Early Treatment Response to Infliximab in Patients Without Surgical History

**FIGURE 3:**
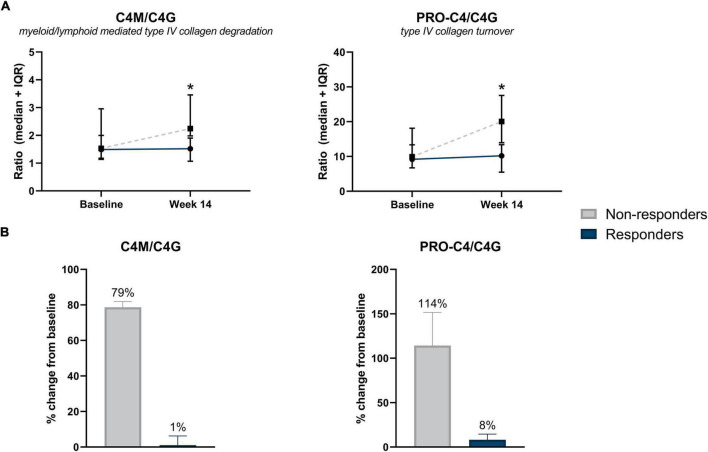
**(A)** Serum biomarker levels in CD patients without surgical history, stratified by response to IFX treatment. Serum levels of type IV collagen ratios C4M/C4G and PRO-C4/C4G were elevated in non-responders at week 14 compared with responders (*P* = 0.035, *P* = 0.020). Significance was calculated using Mann–Whitney *U*-test. Data are shown as median + IQR. **(B)** Percent change from baseline of type IV collagen turnover between responders and non-responders, within CD patients with no history of prior surgery. Non-responders showed a 79 and 114% increase from baseline of type IV collagen turnover ratios C4M/C4G and PRO-C4/C4G, respectively. *P < 0.05.

Serum concentrations of biomarkers in patients with CD without surgical history, stratified by response to IFX are also presented in Table 3. Serum ratios of type IV collagen turnover (C4M/C4G and PRO-C4/C4G) were elevated in non-responders at week 14 compared with responders, with changes from baseline in non-responders being 79 and 114%, respectively (C4M/C4G: 2.25 [2.11, 2.85] vs. 1.52 [1.09, 1.90], *P* = 0.035; PRO-C4/C4G: 20.0 [17.00, 23.80] vs. 10.2 [5.77, 13.01], *P* < 0.05) (Figures 3A,B). ROC statistics revealed that both biomarker ratios could successfully differentiate between responders and non-responders at week 14 (AUC with 95% CI: C4M/C4G 0.87 [0.72–1.0], *P* = 0.035; PRO-C4/C4G 0.91 [0.76–1.0], *P* = 0.020) (Supplementary Table 3). In contrast to patients with a surgical history, a trend was observed within the group where biomarkers of type I, III, and VI collagen degradation were on average elevated in non-responders compared with responders (Table 3).

**FIGURE 4:**
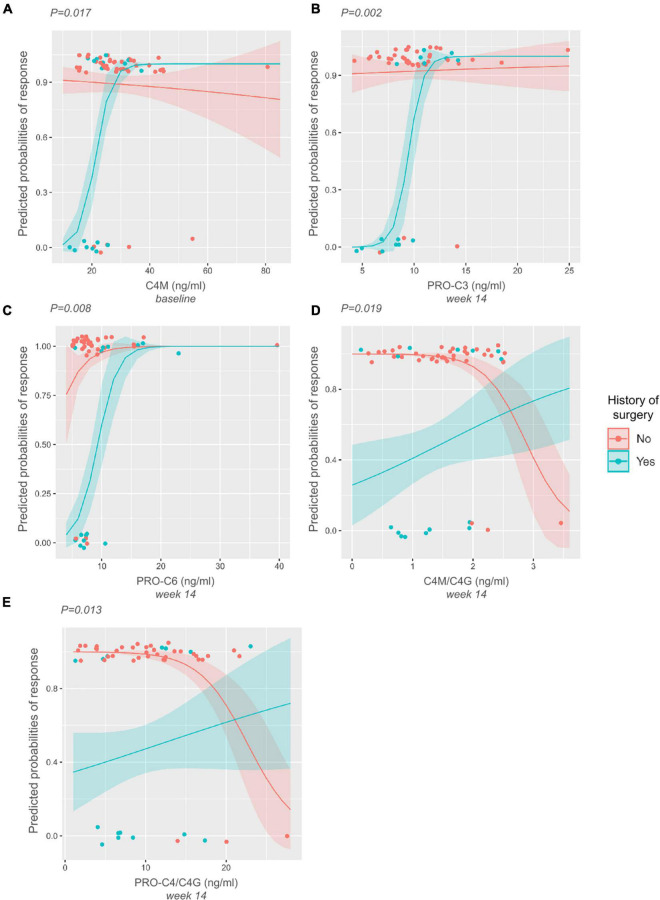
Interaction plots displaying differing biomarker behavior depending on the history-of-surgery status. *P*-values are derived from likelihood-ratio tests depicting the significance of the interaction between the biomarkers and history of prior surgery. **(A–C)** Low baseline levels of C4M, and low levels of PRO-C3 and PRO-C6 at week 14 are indicative of non-response to treatment in patients with surgical history. **(D–E)** Low levels of C4M/C4G and PRO-C4/C4G at week 14 are indicative of response to treatment in patients without surgical history. The opposite can be seen within the group of patients with surgical history, where low levels of these ratios decrease the probability of responding to treatment.

A logistic interaction model identified five biomarkers that showed significantly differing behavior depending on surgical history. Low baseline levels of C4M were indicative of non-response to treatment in patients with surgical history, while no effect was seen in patients without surgical history (*P* < 0.05) (Figure 4A). The same pattern was observed for PRO-C3 and PRO-C6 levels at week 14 (*P* < 0.01) (Figures 4B,C). C4M/C4G and PRO-C4/C4G ratios at week 14 demonstrated contrasting behavior: low values of the turnover ratios were indicative of response in patients without prior surgical history, while low values in patients with surgical history seemed to decrease the probability of patients responding to treatment (both *P* < 0.05) (Figures 4D,E).

## Discussion

Determining the optimal treatment for CD in a timely and non-invasive manner remains a challenge in clinical practice. In this study, we show that serological biomarkers of collagen formation and degradation have promising potential for predicting and monitoring early response to infliximab treatment in patients with CD, which largely depends on patients’ surgical history. We observed that compared with non-responders, patients with a surgical history that responded to IFX treatment demonstrated elevated baseline levels of type IV (C4M) collagen degradation, as well as elevated levels of type VI (C6Ma3) collagen degradation and formation of type III (PRO-C3), and VI collagen (PRO-C6) at week 14 of treatment. Baseline C4M was associated with response to IFX, while C6Ma3, PRO-C3, and PRO-C6 could distinguish between responders and non-responders at week 14. In the group without surgical history, responders to IFX had lower levels of type IV collagen turnover ratios (C4M/C4G, PRO-C4/C4G). These biomarker ratios could also differentiate between responders and non-responders to IFX at week 14. Moreover, we found that clinical laboratory parameters collected at baseline did not seem to be associated with response to treatment.

When looking at patients with surgical history, we noted that the majority had either stricturing or penetrating disease, or in other words, a more complicated disease phenotype. Stricturing disease is characterized by fibrosis, caused by continuous tissue injury and excess ECM deposition, while penetrating disease is characterized by transmural inflammation. It is important to note that patients with penetrating disease usually also present with fibrosis, meaning that in most cases their disease is not confined to an inflammatory phenotype (22–24). In contrast, patients without surgical history mainly present with a luminal disease phenotype or a less complicated phenotype characterized by superficial inflammation of the GI tract without other complications such as strictures or fistulas (25). We observed significantly elevated levels of type I, III, IV, and VI collagen degradation in patients without surgical history, which could be explained by the phenotypic difference just presented between the two groups. In other words, patients with a less complicated disease phenotype, mainly represented by luminal inflammation, have overall higher levels of ECM degradation compared with patients with a more complicated disease phenotype. This has been observed before in a study conducted by van Haaften et al., where patients with CD were grouped by disease behavior according to the Montreal classification (non-stricturing/non-penetrating, stricturing, or penetrating) and biomarkers of collagen formation and degradation were measured. Results showed that the ratio of type III collagen turnover (PRO-C3/C3M) was higher in non-stricturing/non-penetrating disease vs. stricturing and penetrating disease, implying greater ECM turnover activity within the luminal disease phenotype (15).

Serum levels of type IV collagen degradation (C4M) were elevated at baseline in patients with surgical history who eventually responded to IFX treatment at week 14, compared with those who did not respond. A similar trend was observed for several other collagen degradation markers (i.e., C1M, C3M, and C6Ma3). The ability to separate responders from non-responders at baseline, before treatment initiation, is important in clinical practice as it can aid in patient selection and therapeutic decision-making. Given that this patient group represents a more fibrotic phenotype, these results could point to fibrosis resolution in the BM and IM (elevated collagen degradation) within the patients responding to treatment, as effective fibrosis resolution requires degradation and removal of the fibrotic ECM (26). It might be noted that C4M levels are already elevated at baseline. A possible explanation for this could be that responders have increased fibrolytic activity compared with non-responders. One can also think of the two response groups in terms of “cold” and “hot” fibrosis: “Cold” fibrosis represents fibrotic tissue with little to no ECM remodeling activity, thus no fibrolysis, while “hot” fibrosis corresponds to fibrotic tissue with active fibrolysis capable of carrying out normal wound healing. When comparing serum biomarker levels between patients with surgical history vs. those without, another trend was observed with C4M and a few other collagen degradation markers (C1M, C3M, and C6Ma3). Patients with surgical history generally tended to have lower levels of these markers at baseline and after IFX treatment at week 14. This could be explained by the fact that these patients may have had the most prominent fibrotic part of their intestines removed, while patients without surgical history might have some subclinical fibrosis being resolved, leading to elevated levels of collagen degradation. Moreover, these patients will on average have more viable intestinal (fibrotic) tissue compared with patients with surgical history, likely resulting in higher levels of collagen degradation markers. Using these markers to differentiate responders from non-responders after the initiation of treatment provides important clinical value. The neo-epitopes being detected are directly linked to tissue turnover within the intestinal mucosa. Therefore, the biomarkers could be used to monitor tissue healing or the increase in inflammation and fibrosis, offering potential for, i.e., identifying patients whose fibrosis might not be improving regardless of their other symptoms, guiding treatment escalation or a switch to another biologic. This form of monitoring is also both more objective than clinical disease scores, and less invasive than current endoscopic procedures.

In this context, it is worth mentioning the type III collagen formation marker: PRO-C3. We observed elevated levels of PRO-C3 at week 14 in the responders within the group of patients with surgical history compared with non-responders. The fragment being detected is the neo-epitope of the released N-terminal pro-peptide of type III collagen, meaning that it is a marker of true type III collagen formation. It is widely used within the field of liver fibrosis, as it reflects active liver fibrogenesis (27). A healthy reference range has been established for PRO-C3 with a lower limit of 6.1 ng/ml [6.1–6.1] and an upper limit of 14.7 ng/ml [14.0–15.3] (28). In the study at hand, we observed that both responders and non-responders fall within the healthy range, with responders showing a 31% increase in the biomarker at week 14. This observation suggests an active healing response in patients responding to IFX treatment, and may support the hypothesis of “cold” and “hot” fibrosis. These results are also consistent with the literature, which states that as soon as the cause of tissue injury has been eradicated (i.e., with treatment), ECM remodeling proceeds as part of tissue healing and eventually leads to fibrosis resolution (26). Although PRO-C3 is a known marker for liver fibrosis, increased PRO-C3 levels at week 14 from baseline in responders do not resemble a pathological state. Therefore, in the context of IBD, PRO-C3 could potentially serve as a marker for tissue healing or early fibrogenesis as tissue healing and fibrosis are intertwined. Additionally, it has been shown that PRO-C3 is not able to differentiate CD patients with stricturing disease from other Montreal disease behavior phenotypes, suggesting that it is not a feasible marker for fibrosis in CD (15). A similar pattern was seen with PRO-C6, where within the group of patients with surgical history, responders at week 14 had higher levels than non-responders. PRO-C6 is a C-terminal released C5 domain of type VI collagen α3 chain, also known as endotrophin, typically reflecting pro-fibrotic signaling, and it has been shown to be associated with fibrosis stages in non-alcoholic fatty liver disease (NAFLD) (29). It has been reported that patients with CD in clinical remission (HBI <5) have higher serum PRO-C6 levels than patients with active disease (30). Based on these findings, and the results presented in this paper, it can be hypothesized that PRO-C6 reflects a tissue healing response in patients with surgical history responding to IFX treatment. Furthermore, serum levels of type VI collagen degradation (C6Ma3) were elevated in responders at week 14 compared with non-responders, further strengthening the hypothesis that responders to IFX have active ECM remodeling within their fibrotic tissue as a result of proper wound healing.

Two distinct type IV collagen ratios (C4M/C4G and PRO-C4/C4G) were elevated at week 14 in non-responders within the group of patients without surgical history, compared with responders. C4M/C4G is a ratio between serum concentrations of two type IV collagen fragments: one fragment generated by MMP-2, -9, and -12 (C4M), and the other generated by the serine protease Granzyme B (C4G). MMPs are expressed by many different cell types such as granulocytes, monocytes, fibroblasts, and endothelial cells. For example, MMP-9 is known to be the major secretion product of activated monocytes and a component of the specific granules of human neutrophils, while macrophages are known to be the primary cellular source of MMP-12 (31–33). Granzyme B is believed to be exclusively produced by cytotoxic T-cells and NK-cells. It induces the breakdown of type IV collagen, enabling infiltration through the BM (13). It can be concluded that although these two fragments are derived from the same protein, they represent two different forms of inflammation; C4G can be directly related to T-cell activity and infiltration (lymphoid lineage), while C4M is more representative of granulocyte- and monocyte-mediated inflammation (myeloid lineage). By combining these two biomarkers into a ratio, we aimed to gain insight into differing inflammation profiles in relation to IFX treatment. Our results showed that non-responders had higher C4M/C4G ratios at week 14 compared with responders. Therefore, it can be assumed that non-responders have a higher degree of MMP-mediated type IV collagen degradation (C4M) and lower T-cell activity (C4G). In contrast, responders to IFX have lower levels of C4M compared to C4G, meaning they have less myeloid cell-mediated inflammation and greater lymphoid (e.g., T-cell) activity. Similarly, non-responders showed elevated levels of the ratio PRO-C4/C4G at week 14 of therapy, compared with responders, suggesting that non-responders have relatively higher levels of PRO-C4 compared with C4G. PRO-C4 measures an internal fragment of the 7S domain of type IV collagen and is reflective of BM turnover – which may imply that non-responders have higher BM turnover, but lower T-cell activity. These results partially agree with a previous study conducted on patients with CD, where serum levels of collagen turnover were compared between responders and non-responders to anti-TNF therapy, excluding patients with surgical history. The results showed that PRO-C4 and C4M levels were increased in non-responders at week 8, indicating altered type IV collagen turnover and increased MMP-mediated degradation in patients who eventually experienced non-response to anti-TNF treatment (17). However, a note of caution is due when comparing results. In the current study, 62% of patients were in remission at baseline according to the Harvey-Bradshaw Index (HBI), while in the aforementioned study all patients had clinically active disease at baseline (moderate/severe disease). This disease inactivity might affect the change in biomarker levels over time between responders and non-responders. Patients with mild disease do not significantly differ in marker levels at baseline, yet the non-responders progress in their disease over time. Meanwhile, patients with moderate/severe disease already present with differing biomarker levels at baseline and maintain their response profile over time.

**FIGURE 5:**
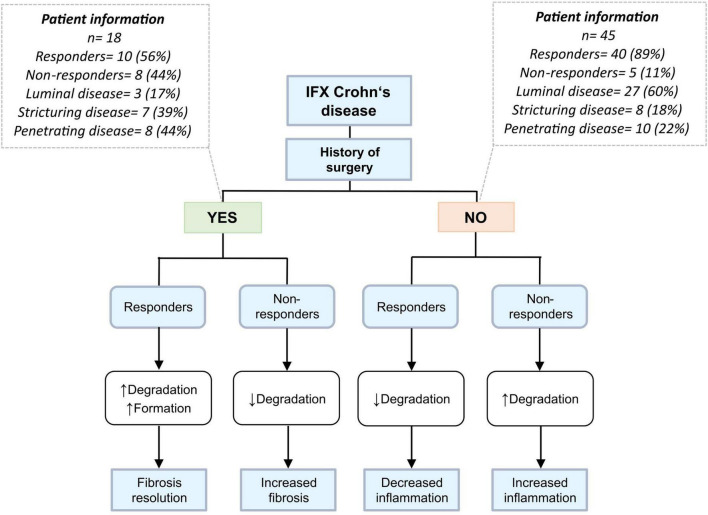
Differing biomarker profiles based on prior history of surgery and disease behavior. Patients were stratified according to prior history of intestinal resection. Eighty-three percent of patients who have undergone prior surgery have stricturing/penetrating disease and thus a more fibrotic phenotype than patients without history of surgery, who mainly have a luminal disease phenotype. Responders within the yes-surgery group would theoretically have elevated levels of degradation markers, a possible result from the breakdown of fibrotic tissue and thus fibrosis resolution – while responders in the no-surgery group have decreased levels of aforementioned biomarkers, suggesting reduced inflammation and improved barrier integrity.

Taken together, responders within the group of patients with surgical history show elevated levels of ECM degradation and formation, indicating fibrosis resolution and tissue healing, which is in harmony with their fibrotic phenotype. These findings are particularly relevant and novel in that patients with CD having prior surgical history are often not investigated independently, i.e., in biomarker discovery studies and clinical trials. Because of the nature of their disease, however, their response profile might look different from patients who have not undergone surgery, and it may therefore be critical to stratify for surgical background. Non-responders within the group of patients without surgical history, many of which were characterized by a luminal disease phenotype, showed elevated levels of biomarker ratios reflecting collagen turnover and degradation of the intestinal mucosa, which may be considered indicative of increased inflammation. This hypothesis is depicted in a flowchart (Figure 5).

Several limitations to this study warrant recognition. First, our sample size was limited, specifically in relation to the group of non-responders, which made it challenging to compare the two response groups. However, this issue was inherent to the study design as the number of responders to IFX generally outweighs that of non-responders with regard to induction therapy. Subsequently, our analyses were stratified by history of prior surgery, which resulted in small subgroups and a decrease in statistical power. A second limitation is the limited availability of disease activity assessment based on the HBI score, both at baseline and week 14. In this study, 30% of HBI scores at baseline were not available, which limited both the assessment of clinical disease activity and the change in activity from baseline to week 14 in relation to the biomarker levels. Furthermore, clinical response to treatment at week 14 was based on a composite of HBI scores, PGA, and steroid-free status, and only PGA and steroid-free status when HBI scores were missing. In this respect, it would have been better to have clinical disease activity defined solely based on standardized HBI scores to enhance comparability to previously performed studies. Another limitation worth noting is missing information on time from surgery to serological measurements. Since ECM remodeling happens over time, differential time duration since surgery may affect these processes. Lastly, for disease activity assessment, we had to rely on clinical and serological assessment of disease activity (i.e., HBI scores, PGA, and CRP values), as data on fecal calprotectin levels or endoscopic disease activity were insufficiently recorded at time of sampling. For future validation studies, these parameters should ideally be readily available to study associations between collagen biomarkers and biochemical and/or endoscopic definitions of treatment response.

## Conclusion

In conclusion, this study demonstrates that baseline levels of a serological biomarker for type IV collagen degradation (C4M) is associated with response to infliximab induction therapy in patients with CD and prior surgical history, and that biomarkers of type III and VI collagen formation, and type VI collagen degradation may be used for monitoring of response at the end of induction therapy in patients with surgical history. Furthermore, biomarker ratios of type IV collagen formation/degradation show potential utility for monitoring treatment response in patients without a surgical history. Our results highlight the potential significance and clinical applicability of serological biomarkers of intestinal collagen turnover for prediction and monitoring of response to infliximab therapy in CD, although future studies are warranted to further validate our findings.

## Data Availability Statement

The datasets generated for this study are available upon reasonable request to the corresponding author.

## Ethics Statement

The studies involving human participants were reviewed and approved by the Institutional Review Board (IRB) of the UMCG (IRB no. 08/338). The patients/participants provided their written informed consent to participate in this study.

## Author Contributions

MA, AB, GD, and JM were involved in conceptualization and study design. GD and JM were responsible for funding acquisition and resources. AB, MA, RL, HD, MV, EF, RW, MK, MP, JM, and GD collected the study data and materials. MA performed the data analysis and data visualization. MA and AB wrote the first draft of the manuscript. All authors contributed to manuscript revision, read, and approved the final version of the manuscript to be submitted for publication.

## Conflict of Interest

MA, MP, MK, and JM are employees of Nordic Bioscience A/S. MK owns stocks in Nordic Bioscience A/S. GD received research grants from Royal DSM and speaker’s fees from Janssen Pharmaceuticals, Takeda, Pfizer, and Abbvie. The remaining authors declare that the research was conducted in the absence of any commercial or financial relationships that could be construed as a potential conflict of interest.

## Publisher’s Note

All claims expressed in this article are solely those of the authors and do not necessarily represent those of their affiliated organizations, or those of the publisher, the editors and the reviewers. Any product that may be evaluated in this article, or claim that may be made by its manufacturer, is not guaranteed or endorsed by the publisher.
