# Supplementary material for: Serological Biomarkers of Intestinal Collagen Turnover Identify Early Response to Infliximab Therapy in Patients With Crohn’s Disease
Source: Front Med (Lausanne). 2022 Jul 12;9:933872. doi: 10.3389/fmed.2022.933872 (PMC9315105; doi:10.3389/fmed.2022.933872)
Supplement: Supplementary file 1 [file Data_Sheet_1.docx]

# **Supplementary Results**


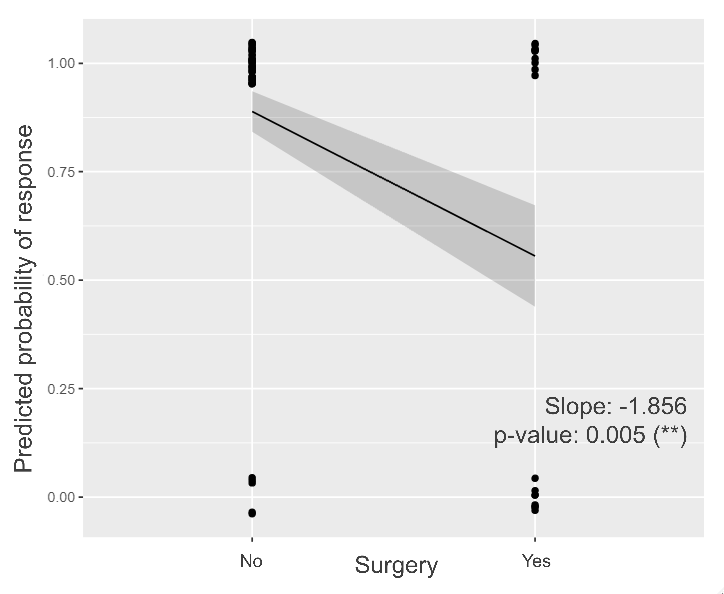


**Supplementary Figure S1.** History of intestinal resection is a strong confounding factor and has a significant effect on response to treatment with a slope of -1.856 (P<0.01, OR = 6.25). Results obtained from a univariable logistic regression model.

**Supplementary Table S1.** Serum biomarker levels stratified by history of surgery, both at baseline and week 14. Values displayed as median with IQR.

| **Biomarker** | | **IFX (n = 63)** | | | | | |
| --- | --- | --- | --- | --- | --- | --- | --- |
|  |  | **History of prior surgery *(yes-surgery)*** n = 18 | | **No history of surgery** ***(no-surgery)*** n = 45 | | **p-value** | |
| ***Baseline*** |  | |  | |  | |  |
| *C1M (ng/ml)* | | 37.5 [31.66, 47.49] | | 61.5 [34.26,104.88] | | 0.075 | |
| ***C3M (ng/ml)*** | | **9.9 [8.44, 11.57]** | | **11.8 [10.00, 14.07]** | | **0.025** | |
| *C3M/PRO-C3 (ng/ml)* | | 1.3 [0.91, 1.41] | | 1.4 [1.16, 1.98] | | 0.107 | |
| *C4G (ng/ml)* | | 18.7 [13.60, 25.10] | | 16.7 [14.08, 22.54] | | 0.670 | |
| ***C4M (ng/ml)*** | | **21.8 [19.04, 24.82]** | | **27.7 [23.07, 32.90]** | | **0.008** | |
| *C4M/C4G (ng/ml)* | | 1.2 [0.95, 1.57] | | 1.5 [1.15, 1.97] | | 0.051 | |
| *C6Ma3 (ng/ml)* | | 0.5 [0.47, 0.53] | | 0.6 [0.48, 0.83] | | 0.140 | |
| *PRO-C3 (ng/ml)* | | 8.6 [7.17, 10.36] | | 8.5 [6.85, 10.39] | | 0.749 | |
| *PRO-C4 (ng/ml)* | | 157.8 [134.44,175.81] | | 178.0 [136.88,233.60] | | 0.294 | |
| *PRO-C4/C4G (ng/ml)* | | 8.6 [5.78, 10.70] | | 9.7 [6.93, 13.18] | | 0.248 | |
| ***PRO-C4/C4M (ng/ml)*** | | **7.2 [6.59, 7.73]** | | **6.6 [5.71, 6.97]** | | **0.020** | |
| *PRO-C6 (ng/ml)* | | 7.6 [6.4, 10.1] | | 7.4 [6.2, 8.9] | | 0.553 | |
| ***Week 14*** |  | |  | |  | |  |
| ***C1M (ng/ml)*** | | **28.3 [23.06, 31.89]** | | **46.0 [26.15, 70.28]** | | **0.022** | |
| ***C3M (ng/ml)*** | | **8.9 [8.20, 10.95]** | | **11.9 [10.64, 14.59]** | | **0.003** | |
| *C3M/PRO-C3 (ng/ml)* | | 1.0 [0.76, 1.52] | | 1.3 [0.96, 1.63] | | 0.275 | |
| *C4G (ng/ml)* | | 16.5 [12.45, 23.27] | | 17.5 [14.10, 20.52] | | 0.765 | |
| ***C4M (ng/ml)*** | | **19.4 [17.84, 21.47]** | | **26.9 [23.93, 31.99]** | | **<0.001** | |
| *C4M/C4G (ng/ml)* | | 1.2 [0.79, 1.94] | | 1.6 [1.14, 2.02] | | 0.311 | |
| *C6Ma3 (ng/ml)* | | 0.5 [0.49, 0.66] | | 0.7 [0.53, 0.75] | | 0.105 | |
| *PRO-C3 (ng/ml)* | | 8.5 [7.60, 10.97] | | 9.5 [8.16, 11.51] | | 0.518 | |
| ***PRO-C4 (ng/ml)*** | | **145.7 [120.06,161.40]** | | **186.2 [147.16,233.68]** | | **0.019** | |
| *PRO-C4/C4G (ng/ml)* | | 6.8 [4.98, 13.65] | | 10.6 [6.16, 14.16] | | 0.469 | |
| *PRO-C4/C4M (ng/ml)* | | 7.2 [6.12, 8.21] | | 6.8 [5.93, 7.44] | | 0.595 | |
| *PRO-C6 (ng/ml)* | | 7.5 [6.4, 10.8] | | 7.5 [6.8, 9.3] | | 0.582 | |

**Supplementary Table S2.** Patient demographic and clinical characteristics, stratified by surgical history and further divided by response to IFX treatment.

|  | Total cohort n = 63 | | | | | | | | |
| --- | --- | --- | --- | --- | --- | --- | --- | --- | --- |
|  | **History of prior surgery**  (yes-surgery) | | | | **No history of surgery**  (no-surgery) | | | | |
|  | **All** (n=18) | **Non-responders** (n=8) | **Responders** (n=10) | ***P* (non-responders vs. responders)** | **All**  (n=45) | **Non-responders** (n=5) | **Responders** (n=40) | ***P* (non-responders vs. responders)** | |
| Age (years) | 43 [37, 56] | 46 [40, 55] | 42 [37, 54] | 0.633 | 29 [25, 42] | 47 [43, 52] | 28 [25, 37] | 0.005 | |
| Gender |  |  |  | >0.999 |  |  |  | 0.634 | |
| *Female* | 9 (50%) | 4 (50%) | 5 (50%) |  | 18 (40%) | 1 (20%) | 17 (42%) |  | |
| *Male* | 9 (50%) | 4 (50%) | 5 (50%) |  | 27 (60%) | 4 (80%) | 23 (57%) |  | |
| BMI (kg/m^2^) | 24.8  [22.2, 28.8] | 28.0  [21.9, 30.2] | 23.3  [22.2, 27.2] | 0.696 | 24.6  [22.1, 29.1] | 28.4  [21.6, 29.9] | 24.4  [22.2, 29.1] | 0.576 | |
| Smoking |  |  |  |  |  |  |  | >0.999 | |
| *No* | 4 (22%) | 1 (12%) | 3 (30%) |  | 16 (36%) | 2 (40%) | 14 (35%) |  | |
| *Previous* | 9 (50%) | 6 (75%) | 3 (30%) |  | 15 (33%) | 2 (40%) | 13 (32%) |  | |
| *Current* | 5 (28%) | 1 (12%) | 4 (40%) |  | 14 (31%) | 1 (20%) | 13 (32%) |  | |
| Montreal classification |  |  |  |  |  |  |  |  | |
| Montreal Age (A) |  |  |  | 0.193 |  |  |  | 0.134 | |
| *A1 (≤ 16 years)* | 2 (11%) | 1 (12%) | 1 (10%) |  | 6 (13%) | 0 (0%) | 6 (15%) |  | |
| *A2 (17-40 years)* | 11 (61%) | 3 (38%) | 8 (80%) |  | 34 (76%) | 3 (60%) | 31 (78%) |  | |
| *A3 (> 40 years)* | 5 (28%) | 4 (50%) | 1 (10%) |  | 5 (11%) | 2 (40%) | 3 (7.5%) |  | |
| Montreal Location (L) |  |  |  | 0.520 |  |  |  | 0.260 | |
| *L1 (ileal disease)* | 10 (56%) | 6 (75%) | 4 (40%) |  | 14 (31%) | 1 (20%) | 13 (32%) |  | |
| *L2 (colonic disease)* | 1 (5.5%) | 0 (0%) | 1 (10%) |  | 4 (9%) | 1 (20%) | 3 (7.5%) |  | |
| *L3 (ileocolonic)* | 6 (33%) | 2 (25%) | 4 (40%) |  | 21 (47%) | 2 (40%) | 19 (48%) |  | |
| *L4 (upper GI disease)* | 1 (5.5%) | 0 (0%) | 1 (10%) |  | 6 (13%) | 1 (20%) | 5 (12.5%) |  | |
| Montreal Behaviour (B) |  |  |  | >0.999 |  |  |  | 0.191 | |
| *B1 (non-stricturing, non-penetrating)* | 3 (17%) | 1 (12%) | 2 (20%) |  | 27 (60%) | 5 (100%) | 22 (55%) |  | |
| *B2 (stricturing)* | 7 (39%) | 3 (38%) | 4 (40%) |  | 8 (18%) | 0 (0%) | 8 (20%) |  | |
| *B3 (penetrating)* | 8 (44%) | 4 (50%) | 4 (40%) |  | 10 (22%) | 0 (0%) | 10 (25%) |  | |
| Montreal Perianal disease (P) | 4 (22%) | 1 (12%) | 3 (30%) | 0.588 | 13 (29%) | 1 (20%) | 12 (30%) | >0.999 | |
| Medication use, n (%) |  |  |  |  |  |  |  |  | |
| *Aminosalicylates* | 0 (0%) | 0 (0%) | 0 (0%) | N/A | 4 (8.9%) | 2 (40%) | 2 (5.0%) | 0.055 | |
| *Steroids* | 6 (33%) | 4 (50%) | 2 (20%) | 0.321 | 12 (27%) | 3 (60%) | 9 (22%) | 0.109 | |
| *Immunosuppressives* | 13 (72%) | 6 (75%) | 7 (70%) | >0.999 | 36 (80%) | 3 (60%) | 33 (82%) | 0.258 | |
| *Prior anti-TNF-α* | 6 (33%) | 3 (38%) | 3 (30%) | >0.999 | 9 (20%) | 1 (20%) | 8 (20%) | 0.156 | |
| *Prior Vedolizumab* | 0 (0%) | 0 (0%) | 0 (0%) |  | 1 (2.2%) | 1 (20%) | 0 (0%) | 0.111 | |
| Surgical history |  |  |  |  |  |  |  |  | |
| *Colectomy* | 4 (22%) | 2 (25%) | 2 (20%) | >0.999 | 0 (0%) | 0 (0%) | 0 (0%) | N/A | |
| *Ileocecal resection* | 15 (83%) | 7 (88%) | 8 (80%) | >0.999 | 0 (0%) | 0 (0%) | 0 (0%) | N/A | |
| Clinical disease activity score | |  |  |  |  |  |  | |  |
| HBI |  |  |  | 0.113 |  |  |  | 0.073 | |
| *Remission (< 5)* | 5 (42%) | 1 (17%) | 4 (67%) |  | 21 (70%) | 2 (40%) | 19 (76%) |  | |
| *Mild disease (5-7)* | 2 (17%) | 2 (33%) | 0 (0%) |  | 4 (13%) | 1 (20%) | 3 (12%) |  | |
| *Moderate disease (8-16)* | 4 (33%) | 3 (50%) | 1 (17%) |  | 4 (13%) | 1 (20%) | 3 (12%) |  | |
| *Severe disease (>16)* | 1 (8.3%) | 0 (0%) | 1 (17%) |  | 1 (3.3%) | 1 (20%) | 0 (0%) |  | |
| Laboratory parameters |  |  |  |  |  |  |  |  | |
| *Hemoglobin (nmol/L)* | 8.15  [7.62, 8.45] | 8.20  [7.52, 8.50] | 7.95  [7.73, 8.28] | >0.929 | 7.95  [7.38, 8.40] | 8.00  [7.40, 8.50] | 7.90  [7.35, 8.35] | 0.985 | |
| *WBC (x10^9^/L)* | 7.50  [6.10, 9.00] | 7.85  [6.08, 9.43] | 6.95  [6.10, 8.30] | 0.594 | 7.60  [6.00, 10.30] | 10.30  [9.8, 10.7] | 7.15  [5.77, 9.18] | 0.108 | |
| *Neutrophil count* | 4.90  [4.01, 6.40] | 4.89  [3.72, 6.48] | 5.16  [4.17, 6.34] | >0.999 | 5.58  [3.90, 7.38] | 7.52  [6.68, 8.27] | 5.30  [3.78, 7.17] | 0.093 | |
| *Eosinophil count* | 0.10  [0.07, 0.14] | 0.11  [0.06, 0.17] | 0.09  [0.08, 0.13] | 0.858 | 0.14  [0.08, 0.20] | 0.24  [0.14, 0.26] | 0.12  [0.07, 0.16] | 0.211 | |
| *CRP (mg/L)* | 3 [2, 5] | 2.5 [0.5, 2.9] | 4.2 [2.6, 7.3] | 0.060 | 8 [4, 15] | 20 [9, 27] | 6 [3, 12] | 0.206 | |
| *Creatinine (µmol/L)* | 69 [63, 82] | 64 [62, 72] | 82 [65, 84] | 0.289 | 63 [58, 69] | 66 [58, 69] | 63 [58, 68] | 0.767 | |
| *eGFR (mL/min/1.73 m^2^)* | 105  [88, 109] | 106  [93, 108] | 91  [84, 111] | 0.773 | 112  [96, 125] | 94  [82, 106] | 114  [98, 127] | 0.064 | |
| *Fecal calprotectin (µg/g)^^^* | 460  [224, 711] | 1860  [740, 2980] | 310  [140, 542.5] | 0.133 | 1,170  [425, 2,630] | 1,170  [650, 2,355] | 1,420  [520, 2,522] | 0.900 | |

**Supplementary Table S3.** Area under the curve analysis of the biomarkers stratified by history of surgery, both at baseline and week 14.

|  | **History of prior surgery** *(Yes-surgery)* | | | | **No history of prior surgery** *(No-surgery)* | | | |
| --- | --- | --- | --- | --- | --- | --- | --- | --- |
| **Biomarker** | AUC (95 % CI) | Sensitivity (%) | Specificity (%) | p-value | AUC (95 % CI) | Sensitivity (%) | Specificity (%) | p-value |
| **Baseline** | | | | |  | | | |
| *C1M* | 0.65 [0.38-0.92] | 40 | 100 | 0.286 | 0.7 [0.47-0.94] | 60 | 80 | 0.139 |
| *C3M* | 0.66 [0.4-0.93] | 50 | 88 | 0.248 | 0.6 [0.39-0.82] | 38 | 100 | 0.448 |
| *C3M/PRO-C3* | 0.49 [0.19-0.78] | 60 | 62 | 0.929 | 0.63 [0.36-0.9] | 35 | 100 | 0.348 |
| *C4G* | 0.6 [0.32-0.88] | 30 | 100 | 0.477 | 0.43 [0.24-0.62] | 25 | 100 | 0.613 |
| ***C4M*** | **0.84 [0.64-1.0]** | **70** | **88** | **0.016** | 0.5 [0.19-0.81] | 97 | 20 | 1.000 |
| *C4M/C4G* | 0.6 [0.31-0.89] | 50 | 88 | 0.477 | 0.57 [0.28-0.87] | 40 | 80 | 0.588 |
| *C6Ma3* | 0.72 [0.47-0.98] | 70 | 75 | 0.110 | 0.54 [0.34-0.74] | 50 | 80 | 0.759 |
| *PRO-C3* | 0.62 [0.35-0.9] | 80 | 50 | 0.374 | 0.37 [0.1-0.64] | 52 | 60 | 0.348 |
| *PRO-C4* | 0.71 [0.46-0.96] | 90 | 50 | 0.131 | 0.57 [0.32-0.81] | 40 | 100 | 0.639 |
| *PRO-C4/C4G* | 0.59 [0.29-0.88] | 60 | 88 | 0.534 | 0.62 [0.41-0.84] | 45 | 100 | 0.367 |
| *PRO-C4/C4M* | 0.4 [0.12-0.68] | 40 | 75 | 0.477 | 0.57 [0.27-0.88] | 88 | 40 | 0.588 |
| *PRO-C6* | 0.78 [0.55-1.0] | 70 | 88 | 0.051 | 0.55 [0.3-0.8] | 28 | 100 | 0.718 |
| **Week 14** | | | | |  | | | |
| *C1M* | 0.67 [0.34-1.0] | 57 | 100 | 0.271 | 0.6 [0.27-0.93] | 44 | 100 | 0.647 |
| *C3M* | 0.48 [0.16-0.81] | 57 | 62 | 0.908 | 0.71 [0.41-1.0] | 53 | 100 | 0.225 |
| *C3M/PRO-C3* | 0.79 [0.52-1.0] | 86 | **75** | 0.064 | 0.59 [0.14-1.0] | 97 | 33 | 0.598 |
| ***C4G*** | 0.54 [0.21-0.87] | 43 | 88 | 0.817 | 0.78 [0.56-1.0] | 58 | 100 | 0.114 |
| *C4M* | 0.64 [0.32-0.96] | 57 | 88 | 0.355 | 0.67 [0.32-1.0] | 50 | 100 | 0.343 |
| ***C4M/C4G*** | 0.62 [0.29-0.96] | 43 | 100 | 0.418 | **0.87 [0.72-1.0]** | **78** | **100** | **0.035** |
| ***C6Ma3*** | **0.83 [0.62-1.0]** | **86** | **75** | **0.032** | 0.65 [0.18-1.0] | 78 | 67 | 0.399 |
| ***PRO-C3*** | **0.95 [0.83-1.0]** | **86** | **100** | **0.004** | 0.44 [0.0-0.9] | 89 | 33 | 0.752 |
| *PRO-C4* | 0.57 [0.25-0.9] | 29 | 100 | 0.643 | 0.73 [0.3-1.0] | 89 | 67 | 0.188 |
| ***PRO-C4/C4G*** | 0.55 [0.22-0.88] | 57 | 75 | 0.728 | **0.91 [0.76-1.0]** | **78** | **100** | **0.020** |
| *PRO-C4/C4M* | 0.46 [0.13-0.79] | 100 | 25 | 0.817 | 0.7 [0.29-1.0] | 81 | 67 | 0.246 |
| ***PRO-C6*** | **0.82 [0.54-1.0]** | **89** | **88** | **0.037** | 0.69 [0.44-0.93] | 53 | 100 | 0.292 |

**Supplementary Table S4.** Optimal cut-off values for each biomarker at baseline and week 14, determined by Youden’s *J* statistics on respective ROC curves.

| **Biomarker** | **Cut-off (ng/ml)** | |
| --- | --- | --- |
| **Baseline** | History of prior surgery | No history of prior surgery |
| *C1M* | 48.1 | 68.3 |
| *C3M* | 10.7 | 11.0 |
| *C3M/PRO-C3^a^* | 1.3 | 1.2 |
| *C4G* | 28.0 | 13.5 |
| *C4M* | 22.7 | 49.7 |
| *C4M/C4G^a^* | 1.5 | 1.3 |
| *C6Ma3* | 0.5 | 0.59 |
| *PRO-C3* | 7.5 | 8.5 |
| *PRO-C4* | 135.3 | 155.1 |
| *PROC4/C4G^a^* | 9.6 | 7.5 |
| *PRO-C4/C4M^a^* | 7.6 | 11.0 |
| *PRO-C6* | 7.8 | 6.2 |
| **Week 14** | History of prior surgery | No history of prior surgery |
| *C1M* | 31.9 | 40.3 |
| *C3M* | 9.4 | 11.9 |
| *C3M/PRO-C3^a^* | 2.0 | 2.5 |
| *C4G* | 23.3 | 11.4 |
| *C4M* | 21.4 | 26.6 |
| *C4M/C4G^a^* | 2.0 | 1.9 |
| *C6Ma3* | 0.52 | 0.75 |
| *PRO-C3* | 10.3 | 13.7 |
| *PRO-C4* | 193.5 | 266.0 |
| *PRO-C4/C4G^a^* | 10.2 | 13.8 |
| *PRO-C4/C4M^a^* | 5.3 | 7.8 |
| *PRO-C6* | 8.7 | 5.4 |

^a^Biomarker ratios with no concentration unit.
